# Correlation between Severity of Idiopathic Epiretinal Membrane and Irvine–Gass Syndrome

**DOI:** 10.3390/jpm13091341

**Published:** 2023-08-30

**Authors:** Jee Hyun Jeong, Kyung Tae Kang, You Hyun Lee, Yu Cheol Kim

**Affiliations:** Department of Ophthalmology, School of Medicine, Keimyung University, Daegu 42601, Republic of Korea; porili@hanmail.net (J.H.J.); kkt0604@dsmc.or.kr (K.T.K.); dyb7535@hanmail.net (Y.H.L.)

**Keywords:** ectopic inner foveal layer, idiopathic epiretinal membrane, Irvine–Gass syndrome, optical coherence tomography, postoperative cystoid macular edema

## Abstract

A higher risk of pseudophakic cystoid macular edema (PCME) has been reported in patients with preoperative idiopathic epiretinal membrane (ERM); however, whether the formation of PCME depends on the grade of ERM has not been well established. We conducted a retrospective case–control study of 87 eyes of 78 patients who were preoperatively diagnosed with idiopathic ERM and had undergone cataract surgery. Patients were divided into two groups: PCME and non-PCME groups. After cataract surgery, the ERM status was graded using the Gass and Govetto classifications. Both the central macular thickness (CMT) and ERM grade increased after surgery, and higher preoperative CMT and ERM grades were found in the PCME group. The association between higher-grade ERM and the development of PCME was significant in the Govetto classification (grade 2, odds ratio (OR): 3.13; grade 3, OR: 3.93; and grade 4, OR: 16.07). The study results indicate that close attention should be given to patients with ERM with the presence of an ectopic inner foveal layer before cataract surgery.

## 1. Introduction

Irvine–Gass syndrome, also known as pseudophakic cystoid macular edema (PCME), is a condition characterized by the formation of cystoid macular edema (CME) that may develop after uneventful cataract surgery. This common complication has the potential to cause visual impairment after surgery. The signs of PCME typically manifest approximately 4–6 weeks after cataract surgery [1]. The reported incidence of clinically significant PCME varies from 0.6% to 2.6%, while subclinical PCME, which is detected using fundus fluorescein angiography or optical coherence tomography (OCT), occurs in approximately 10–20% of cases [1,2,3,4]. The introduction of phacoemulsification, a modern cataract surgery technique, has led to a decrease in the incidence of PCME compared to the previous extracapsular cataract extraction technique. A recent study by Chu et al. [5] reported the mean incidence of PCME to be 1.17% in the eyes of patients without diabetes that had undergone cataract surgery using modern phacoemulsification techniques. While the exact pathology of PCME is not yet fully understood, it can largely be explained by two theories [6]. The first is the “the inflammation theory”, which suggests disruption of the blood–retina barrier by inflammatory mediators and the vascular endothelial growth factor (VEGF) after cataract surgery. This results in the accumulation of perifoveal intraretinal fluid because of the increased permeability of the perifoveal capillaries. The second is the “vitreous traction theory”, which suggests direct macular traction of the vitreous humor at the anterior segment structures. There are several risk factors for PCME. Diabetes is the most significant one, with a reported four-fold-increased risk compared to patients without diabetes [5]. Uveitis, previous retinal vein occlusion, previous retinal detachment repair, epiretinal membrane (ERM), and intraoperative events, such as increased phacoemulsification time, energy, and posterior capsule rupture during surgery, were also found to increase this risk [1,5,7].

ERM is an avascular proliferative fibro-cellular membrane that develops on the inner surface of the retina. It can cause macular wrinkling, traction, and edema. Idiopathic ERM generally occurs after posterior vitreous detachment and fibroblast activation, and patients with idiopathic ERM are found to have higher concentrations of fibrotic and inflammatory cytokines [8]. ERM can progress with age, but it can also develop as a secondary condition resulting from intraocular surgery, retinal vascular disease, inflammation, or trauma [9,10]. Previously, the diagnosis of ERM relied solely on clinical findings through slit-lamp and fundus examination. In 1987, Gass proposed the first ERM classification system based on fundus examination. According to this system, grade 0 corresponds to cellophane maculopathy, which is a translucent membrane without distortion of the inner retina with no symptoms. Grade 1 refers to crinkled cellophane maculopathy, characterized by fine retinal folds, wrinkling of the inner retina, and symptoms of metamorphopsia. Grade 2 represents a macular pucker of thick opaque membranes with full-thickness retinal distortion [11]. However, with the advent of spectral-domain OCT, the diagnosis of ERM has primarily relied on this imaging modality owing to its advantages in early detection and severity staging [12,13]. Various studies have highlighted the importance of inner retinal layer thickness in predicting visual prognosis in patients with ERM [14,15,16,17]. Govetto et al. [18] confirmed the sensitivity of the inner retinal layers of the macula to traction stress and proposed a novel ERM staging scheme using OCT. In this staging scheme, ERM is classified into four stages using the ectopic inner foveal layer (EIFL) extending from the inner nuclear layer and the inner plexiform layer across the central fovea as key components. Stage 1 is characterized by the presence of a foveal pit and well-defined retinal layers, whereas, in stage 2, the foveal pit is absent, but the retinal layers are still well defined. In stage 3, an EIFL is present, and in stage 4, the retinal layers are disrupted.

Multiple studies [1,4,5] have reported a notable elevation in the incidence of PCME when patients present with pre-existing ERM prior to undergoing cataract surgery. However, the extent to which the severity of ERM correlates with PCME development is not yet well established. Therefore, the purpose of this study was to evaluate the correlation between ERM severity, as determined through fundus findings and OCT imaging, and the development of PCME.

## 2. Materials and Methods

This retrospective, single-center study aimed to analyze the clinical records of a significant number of patients to investigate the association between idiopathic ERM and the development of PCME following cataract surgery. The study focused on 579 patients who underwent cataract surgery at Keimyung University Dongsan Hospital between August 2018 and March 2022. The study was conducted in accordance with the ethical guidelines and approved by the Institutional Review Board of Keimyung University Dongsan Hospital (approval no. 2022-05-046), ensuring compliance with the principles of the Declaration of Helsinki. Owing to the retrospective nature of this study, the requirement for informed consent was waived.

Patients with idiopathic ERM who had undergone uneventful cataract surgery without any other combined surgery were included. The study required recorded OCT images both before and after cataract surgery, within a timeframe of 3 months. In contrast, patients with a history of previous vitrectomy and retinal vascular diseases, such as exudative macular degeneration, retinal vein occlusion, diabetic retinopathy, vasculitis, or uveitis, were excluded. Additionally, patients with high myopia (axial length ≥ 26 mm) or any other condition that could have caused secondary ERM were not included. If both eyes of a patient met the inclusion and exclusion criteria, both eyes were included in the study.

Eighty-seven eyes were divided into two groups: the PCME group, which consisted of patients who developed PCME on OCT after cataract surgery, and the non-PCME group. The researchers collected comprehensive information from the patient’s medical records, including previous ophthalmologic history, age, and sex. Records of preoperative and postoperative visual acuity, best-corrected visual acuity (BCVA), ERM status as determined under slit-lamp biomicroscopy and via OCT, central macular thickness (CMT), and the presence of CME were also obtained. The researchers used a swept-source OCT device (DRI-OCT Triton; Topcon Inc., Tokyo, Japan) to obtain OCT images for the identification of PCME, CMT, and ERM. In cases where multiple OCT images were captured during the 3-month follow-up period after surgery, the image exhibiting the highest CMT, or, if PCME was detected, the one exhibiting the most active PCME, was chosen for analysis. CMT was measured at a central 1 mm area, defined as the distance between the internal limiting membrane and the inner border of the retinal pigment epithelium, using a topographic map with an Early Treatment Diabetic Retinopathy Study grid. PCME was defined as the presence of newly emerging extracellular cystic spaces in the macular area on OCT imaging. Thickening in the inner or outer nuclear layers without cystic changes was not counted as PCME. Furthermore, the researchers also assessed the preoperative and postoperative ERM status using two different classifications, the “Gass classification [11]” and the “Govetto classification [18]”, with the presence of an EIFL staged by OCT as a key component.

### 2.1. Surgical Procedure

The surgical procedures were performed by three experienced retinal surgeons (YCK, KKT, and YHL). Prior to surgery, patients were instructed to apply an antibiotic ophthalmic solution, either levofloxacin (Cravit 1.5% ophthalmic solution, Santen Pharmaceutical, Co., Ltd., Osaka, Japan) or moxifloxacin (Vigamox 0.5% ophthalmic solution, Alcon Laboratories, Inc., Fort Worth, TX, USA), for a duration of 3 days. The cataract surgery itself involved several steps performed with precision and care. A 2.2 mm clear corneal incision was made, followed by continuous curvilinear capsulorhexis, hydrodissection, hydrodelineation, phacoemulsification of the nucleus, and aspiration of the residual cortex. Subsequently, an acrylic intraocular lens was implanted in the posterior chamber, and any remaining viscoelastics were carefully removed. Postsurgery, a regimen of antibiotic ophthalmic solution was administered to all patients to prevent potential infections. They were prescribed either levofloxacin (Cravit 1.5% ophthalmic solution, Santen Pharmaceutical, Co., Ltd., Osaka, Japan) or moxifloxacin (Vigamox 0.5% ophthalmic solution, Alcon Laboratories, Inc., Fort Worth, Texas, USA). Additionally, patients were instructed to apply prednisolone acetate ophthalmic solution 1% (Predforte, Allergan, Inc., Irvine, CA, USA) every 2 h, excluding sleep time, for the first week following surgery. All surgeries were performed using the same machine (Centurion, Alcon Laboratories, Inc.). This consistency in equipment ensured standardized surgical techniques and outcomes for all patients included in the study.

### 2.2. Statistical Analysis

All data were analyzed using IBM SPSS Statistics ver. 23.0 (IBM Corp., Armonk, NY, USA). Statistical significance was set at *p* < 0.05.

The comparison between preoperative and postoperative values was performed using paired *t*-tests and Wilcoxon signed rank tests, and the comparison between the non-PCME and PCME groups was performed with independent sample t-tests for continuous variables and chi-square tests for categorical variables. Normality testing was conducted prior to the analysis. Logistic regression analysis was used to determine the relationship between ERM staging and the presence of PCME.

## 3. Results

A total of 579 patients diagnosed with ERM who underwent cataract surgery were initially reviewed. Among them, 391 patients were excluded as they had undergone combined surgery or had a history of vitrectomy owing to other retinal diseases, including ERM; in addition, 128 patients were excluded as they had retinal vascular diseases, and 55 patients were excluded as their OCT images were missing at 3 months before and after cataract surgery. In total, 87 eyes of 78 patients were enrolled in the study (sex: 56 (64.4%) women and 31 (35.6%) men); the average age of the patients was 73.41 ± 8.66 years. Twenty-nine (33.3%) patients were diagnosed with diabetes. Using OCT examination, 48 (55.2%) eyes were classified into the PCME group and 39 (44.8%) into the non-PCME group. The demographic data of the enrolled patients are presented in Table 1.

Postoperative variables were measured 1.37 ± 0.88 (range: 0.17–3.2) months after surgery. Both the CMT and ERM grades increased after cataract surgery. CMT increased from 286 ± 70.0 μm preoperatively to 310 ± 77.7 μm postoperatively (*p* < 0.0001), with a significant increase being identified both in the PCME and the non-PCME group (*p* < 0.0001 in each case). The ERM grade exhibited a significant overall elevation as per the Gass classification (*p* < 0.0001): six cases progressed from grade 0 to 1, eight cases advanced from grade 1 to 2, and one case escalated from grade 0 to 2. Similarly, based on the Govetto classification (*p* < 0.0001), six cases advanced from grade 1 to 2, three cases progressed from grade 2 to 3, and four cases elevated from grade 3 to 4, as presented in Table 2.

When comparing the non-PCME and PCME groups, the latter had a higher preoperative CMT of 301 ± 10.0 μm (*p* = 0.023) and higher ERM grade according to both the Gass (*p* = 0.002) and Govetto (*p* = 0.004) classifications (Table 3). There were no significant differences in age, sex, or diabetes between the groups.

The association between the ERM grade and PCME development was determined using univariate logistic regression analysis (Table 4). In the Gass classification, grade 2 showed a significant increase in PCME with an odds ratio (OR) of 5.99 (*p* = 0.0012), while grade 1 showed an OR of 1.74 with no statistical significance (*p* = 0.373). In the Govetto classification, significant associations were found for all grades: grade 2 (OR: 3.13, *p* = 0.0120), grade 3 (OR: 3.93, *p* = 0.0310), and grade 4 (OR: 16.07, *p* = 0.0120).

## 4. Discussion

Although there are many relevant risk factors for the formation of PCME, this study aimed to investigate the role of pre-existing ERM as a risk factor for the development of PCME, excluding other potential factors. Previous research has consistently demonstrated that ERM is a predictive factor for PCME formation, thus motivating the current study to build upon these findings [1,4,5]. Chu et al. [5] reported a 5.60-fold-increased risk of PCME in the presence of ERM. Henderson et al. [1] stated that patients with preoperative ERM had an incidence rate of PCME of 7.7%. Schaub et al. [4] identified a 2.98-fold-increased risk of PCME in eyes with pre-existing ERM, while a history of pars plana vitrectomy was associated with a 3.58-fold-increased risk.

The findings of this study reveal that the preoperative CMT and ERM grades, according to both the Gass and Govetto classifications, were higher in the PCME detection group. The observed association between higher preoperative CMT and PCME aligns with the results of previous studies, which have indicated a greater incidence of PCME in eyes with elevated preoperative CMT [19]. Furthermore, the OR for developing PCME increased with the increasing severity of ERM. Only grade 1 in the Gass classification showed a higher OR than grade 0 at 1.74, with no statistical significance. These findings suggest a correlation between the development of PCME and the severity of ERM. This correlation appeared more pronounced when using the Govetto classification based on OCT rather than the Gass classification based on fundus photographs. The Govetto classification, which assesses microstructural changes using OCT, exhibited a higher OR as ERM grading increased, and the differences were statistically significant for all grades. In both the Gass and Govetto classifications, no patients with PCME exhibited lower grades of ERM compared to their preoperative grades. In the conventional Gass grading system, ERM is recognized only by clinical examination, thereby limiting the grading of ERM in patients with a moderate-to-severe cataract status [7]. However, owing to improved imaging technology, OCT can be used for this purpose, and the latest grading system developed by Govetto et al. [18] identifies microstructural changes based on OCT. This grading system for detecting the EIFL has been found to be effective for grading retinal damage and visual loss in eyes with ERM [20,21].

A study conducted by Vallejo-Garcia et al. [22] reported no statistically significant correlation between the stage of ERM and the development of CME. Their investigation utilized the Govetto classification and analyzed postoperative variables 1 month after cataract surgery. The selection criteria for their study focused on patients who underwent sequential cataract and idiopathic ERM surgeries, indicating that these patients would have had severe ERM. Only 8.9% of the patients were classified as being at stage 1, and 42.9% and 39.3% of them were classified as being at stage 2 and 3, respectively. Our selected patients mostly had stage 1 disease (44.8%), which led to contradictory results. In patients with a higher ERM grade, intraretinal cystic changes are often observed, posing a challenge in distinguishing active edema from tractive components. Consequently, preoperative lower-grade ERM may serve as a more accurate predictor for the formation of PCME compared to cases with higher-grade ERM and intraretinal cysts.

Another noteworthy finding in our study was that the ERM grade, according to both the Gass and Govetto classifications, increased after cataract surgery. The influence of anterior segment conditions on ERM formation remains controversial, although several studies have identified cataract surgery as a major surgical cause of ERM [23,24]. Jahn et al. [25] demonstrated an increased prevalence of idiopathic ERM at 6 months after extracapsular cataract extraction. Contrary to our findings, Hayashi et al. [9] concluded that idiopathic ERM was not accelerated by small-incision phacoemulsification. In our study, the increase in the Gass classification was 0 to 1 in seven patients, 1 to 2 in eight patients, and stage 0 to 2 in only one patient. These findings may also be the result of hazy fundoscopic examinations due to cataracts, potentially leading to an underestimation of the ERM stage. In the Govetto classification, 6 out of 14 patients displayed an increase from stage 1 to 2, which may be attributed to acquired CME, as evidenced by the absence of a foveal pit on OCT. Nonetheless, three patients showed an increase from stage 2 to 3, and four patients showed an increase from stage 3 to 4. These findings imply the progression of ERM after cataract surgery. Concerning the pathophysiology of ERM formation, the anteroposterior movement of the vitreous during cataract surgery and inflammation after surgery would result in the progression of ERM. It has also been reported that eyes with partial posterior vitreous detachment have a three-times-higher risk of ERM progression after cataract surgery [26].

Concerning the treatment of patients with PCME, the majority of cases resolved spontaneously or with the use of topical non-steroidal anti-inflammatory drugs (NSAIDs). Effective outcomes have also been observed with the administration of anti-VEGF agents, as well as intravitreal or posterior sub-Tenon corticosteroid injections [1,27,28,29,30]. However, it is important to note that, in some cases, persistent edema can occur, resulting in a decline in visual acuity and permanent vision loss, even after the resolution of the edema. Hunter et al. [31] reported that 26.8% of patients with PCME did not return to 20/20 visual acuity following the resolution of macular edema. However, there is still no consensus on the most appropriate treatment for patients with PCME who present preoperative pre-existing risk factors, such as ERM. In a previous study, the effectiveness of combining oral steroids and topical NSAIDs was examined, but no significant improvement in patient outcomes was observed [32]. Furthermore, for patients requiring management of ERM, it may be worth considering performing PPV prior to cataract surgery. A previous study showed that performing PPV with membrane peeling before cataract surgery can lead to better visual outcomes and comparable rates of PCME occurrence [19]. In our study, involving a cohort of 48 patients with PCME, 9 individuals were lost to follow up. Among the remaining patients, 12 cases experienced spontaneous resolution, 19 cases were treated with topical NSAIDs, and 8 underwent treatment with intravitreal corticosteroid injections. Notably, six of these cases displayed inadequate response, necessitating vitrectomy within 6 months following cataract surgery. These results underscore that the origins of PCME in patients with pre-existing ERM can be attributed to both postsurgical inflammatory component and the tractional component. Therefore, additional research is necessary to ascertain the optimal treatment approach for patients with pre-existing ERM.

It has been reported that patients with diabetes have a significantly increased risk of postoperative edema [5]. In this study, only patients with diabetic retinopathy were excluded, while patients with diabetes mellitus (DM) were not excluded. Segura et al. [33] conducted a study comparing macular thickness changes after uncomplicated phacoemulsification surgery, featuring two groups: a DM group without diabetic retinopathy and a non-DM group. The authors found increased macular thickness in both groups, yet discerned no significant differences between them. Our study aligns with these findings, where out of a total of 29 diagnosed DM patients, 20 exhibited PCME while 9 did not. Furthermore, we discovered no significant correlation between the presence of DM and PCME formation (*p* = 0.067).

This study had some limitations. First, with the advent of technology, microcystic macular edema with increased CMT was also counted as PCME, which might have been the reason for the high PCME occurrence rate. However, whether newly emerged CME can be observed as a postsurgical inflammatory component or as a traction component resulting from ERM progression remains controversial. Second, this study was not designed to compare the results with the natural course of ERM. Idiopathic ERM can exhibit intraretinal cystic changes. Therefore, further studies focused on idiopathic ERM without cataract surgery should be conducted. Third, because of the multicollinearity between the Gass and Govetto classifications, there was a limitation in comparing the relevance of the two different grading systems in PCME development.

## 5. Conclusions

By analyzing the collected data, this study aimed to explore the relationship between ERM severity, as determined by the two classification systems, and the development of PCME following cataract surgery. The risk of developing PCME was found to be increased with ERM severity. Its relevance was more evident when the Govetto classification based on OCT was employed. In recent years, several studies have reported inner retinal status as a prognostic factor in ERM surgery, and that eyes without EIFL formation have a better postoperative BCVA [14,34]. The findings of this study have the potential to contribute to a better understanding of the risk factors and mechanisms associated with PCME. Based on the results of our study, closer attention should be paid to patients with ERMs before cataract surgery, especially in eyes with an EIFL.

## Figures and Tables

**Table 1:** Baseline data characteristics.

	Total (*n* = 87)
Sex (Female:Male) (%)	31 (35.6):56 (64.4)
Age, years	73.41 ± 8.66
BCVA	0.43 ± 0.23
Diabetes	29 (33.3)
ERM staging—Gass classification	
0	25 (28.7)
1	20 (23.0)
2	42 (48.3)
ERM staging—Govetto classification	
1	39 (44.8)
2	19 (21.6)
3	19 (21.6)
4	10 (11.5)
CMT (μm)	286.13 ± 69.97
CME	48 (55.2)

Values are presented as numbers (%) or means ± standard deviations. Abbreviations: ERM, epiretinal membrane; CME, cystoid macular edema; CMT, central macular thickness.

**Table 2:** Preoperative and postoperative values.

	Preoperative	Postoperative	*p*-Value
BCVA	0.43 ± 0.23	0.61 ± 0.26	<0.0001
CMT	286 ± 70.0	310 ± 77.7	<0.0001
PCME	301.35 ± 69.37	333.40 ± 74.70	<0.0001
Non-PCME	267.38 ± 66.89	283.72 ± 72.12	<0.0001
ERM staging—Gass classification (%)			<0.0001
0	25 (28.7)	18 (20.7)	
1	20 (23.0)	18 (20.7)	
2	42 (48.3)	51 (58.6)	
ERM staging—Govetto classification (%)			<0.0001
1	39 (44.8)	33 (37.9)	
2	19 (21.6)	22 (25.3)	
3	19 (21.6)	17 (19.5)	
4	10 (11.5)	15 (17.2)	

Values are presented as means ± standard deviations. ERM staging was measured using Wilcoxon signed-rank test. Abbreviations: CMT, central macular thickness; ERM, epiretinal membrane; PCME, pseudophakic cystoid macular edema.

**Table 3:** Characteristics of groups with and without PCME.

	PCME Group (n = 48)	Non-PCME Group (n = 39)	*p*-Value
Age	73.6 ± 7.82	73.4 ± 9.64	0.929
Sex (F:M)	31:17	25:14	0.963
Diabetes (%)	20 (41.7)	9 (23.1)	0.067
Preop CMT	301 ± 10.0	267 ± 10.7	0.023
Preop ERM staging—Gass classification (%)			0.002
0	8 (16.7)	17 (43.6)	
1	9 (18.8)	11 (28.2)	
2	31 (64.6)	11 (28.2)	
Preop ERM staging—Govetto classification (%)			0.004
1	14 (29.2)	25 (64.1)	
2	11 (22.9)	5 (12.8)	
3	14 (29.2)	8 (20.5)	
4	9 (18.8)	1 (2.6)	

Age and preop CMT were measured with paired *t*-tests. Sex, diabetes, and preop ERM staging were measured with chi-square tests. Values are presented as means ± standard deviations. Abbreviations: CMT, central macular thickness; ERM, epiretinal membrane; PCME, pseudophakic cystoid macular edema.

**Table 4:** Relationship between ERM staging and occurrence of PCME.

	Crude Odds Ratio		
	OR	95% CI	*p*-Value
	ERM staging—Gass classification		
0	1		
1	1.74	0.52–5.87	0.373
2	5.99	2.02–17.74	0.001
	ERM staging—Govetto classification		
1	1		
2	3.13	1.05–9.27	0.04
3	3.93	1.13–13.62	0.031
4	16.07	1.84–140.35	0.012

Abbreviations: CMT, central macular thickness; ERM, epiretinal membrane; PCME, pseudophakic cystoid macular edema.

## Data Availability

Data are available upon request owing to privacy or ethical restrictions.
